# Improved Properties of Metallocene Polyethylene/Poly(ethylene terephthalate) Blends Processed by an Innovative Eccentric Rotor Extruder

**DOI:** 10.3390/polym12030585

**Published:** 2020-03-05

**Authors:** He-Zhi He, Feng Xue, Bin Xue, Shi-Ming Liu, Zhao-Xia Huang, He Zhang

**Affiliations:** 1 National Engineering Research Center of Novel Equipment for Polymer Processing, School of Mechanical and Automotive Engineering, South China University of Technology, Guangzhou 510640, China; xuexice@163.com (F.X.); breeze_z3@163.com (S.-M.L.); mehuangzx@scut.edu.cn (Z.-X.H.); zhanghe@scut.edu.cn (H.Z.); 2 Key Laboratory of Polymer Processing Engineering, Ministry of Education, School of Mechanical and Automotive Engineering, South China University of Technology, Guangzhou 510640, China; 3 Guangdong Provincial Key Laboratory of Technique and Equipment for Macromolecular Advanced Manufacturing, School of Mechanical and Automotive Engineering, South China University of Technology, Guangzhou 510640, China

**Keywords:** metallocene polyethylene (m-PE), poly(ethylene terephthalate) (PET), eccentric rotor extruder (ERE), twin-screw extruder (TSE), compatibility

## Abstract

In this investigation, metallocene polyethylene (m-PE) was melt blended with poly(ethylene terephthalate) (PET) with an effort to achieve improved mechanical properties using a novel eccentric rotor extruder (ERE) without the addition of any compatibilizers. The phase morphology, rheological properties, crystallization behavior, and mechanical properties of the fabricated blends were carefully studied to explore the effect of the elongational flow field on the dispersion and mixing of PET in the m-PE matrix and the interface of the two immiscible polymers. For comparison, a conventional twin-screw extruder (TSE) was used to prepare the same blends as references using the same processing condition. It shows that the elongational flow field in ERE is much more effective to disperse the immiscible PET in the m-PE matrix with a smaller particle size and a narrower particle size distribution, compared to the shear flow field in TSE. A compatibilizer, ethylene-co-methyl acrylate-co-glycidyl methacrylate (E-MA-GMA), was added to the m-PE/PET blends during the processing using TSE and ERE. It was observed that the improvement of the tensile property by adding the compatibilizer is marginal for the m-PE/PET blends processed using ERE, which indirectly proves the high effectiveness of the elongational flow field on the enhancement of the dispersion and mixing of PET in the m-PE matrix and the interface interaction.

## 1. Introduction

Metallocene polyethylene (m-PE) is a type of PE which is synthesized under the catalysis of metallocene and was first industrialized by Exxon in 1991 [1]. Benefiting from narrow molecular weight distribution and uniform comonomer distribution, compared with the traditional linear low-density polyethylene (LLDPE), the m-PE has a large elongation at break, excellent impact resistance, good tear strength, and puncture resistance [2]. However, the low strength, low resistance to heat and poor processability of m-PE seriously limit its practical applications. Therefore, the current studies about m-PE are focusing on how to improve the strength, heat resistance, and processing properties by means of blending and modifying. The method of blending with other polymers is simple and effective, and it is capable of combining the merits of different polymers in one material, greatly extending the application of m-PE.

Polyethylene terephthalate (PET) is a common engineering plastic. Owing to the rigid benzene rings in its molecular structure, it has high strength and good heat resistance. Most importantly, it is inexpensive and has been widely applied all over the world. Since PET has a relatively low viscosity during the melt process, it has been employed to improve not only the processing performance of the highly viscous m-PE but also the strength and heat resistance of materials based on m-PE. By blending these two polymers, it has the potential to prepare the material with balanced properties, extending the application for both plastics [3,4]. 

However, attributed to the intrinsic nature of the two polymers, that is, m-PE is a non-polar polyolefin but PET is a polyester, m-PE and PET are thermodynamically immiscible with each other. The performance of the blends obtained by conventional melt blending is relatively poor, thus, it is not beneficial to its practical application. To date, the commonly used method is to add a compatibilizer which is suitable for the blends [5,6]. The added compatibilizer can significantly reduce the interfacial tension of the two immiscible phases, making the discontinuous phase more easily broken and evenly dispersed, and thus significantly improving the interfacial adhesion [7,8]. Pracella et al. [9]. investigated the effects of different compatibilizers on the morphology, melt behavior, rheological behavior, thermal behavior, and dynamic mechanical properties of PET/polyolefin blends, finding that an effective compatibilizer can obviously improve the mechanical properties of this blend.

Zhang et al. [10,11,12]. conducted a series of studies on the recycled PET (R-PET) and PE blends. The styrene ethylene/butylene styrene grafted maleic anhydride (SEBS-g-MAH) compatibilizer was first used to study the compatibilization of R-PET/linear low density polyethylene (LLDPE) blends. It was found that the addition of the compatibilizer can significantly improve the compatibility and the corresponding tensile properties. With increasing the SEBS-g-MAH content, the tensile strength and elongation at break of the blend increase gradually, although the flexural strength decreases. When the content of the added compatibilizer was 10 wt% of the total weight of the blend, the impact strength and the elongation at break of the blend were as high as 147.3 kJ/m2 and 267.5%, respectively, which are much higher than those without the compatibilizer. It was also found that the addition of SEBS-g-MAH firstly increases and then decreases the fluidity of the blending system. Subsequently, a compatibilizer based on the linear low density polyethylene grafted maleic anhydride (LLDPE-g-MAH) was used to study its effect on the structure and properties of the materials. Experiments show that LLDPE-g-MAH can significantly improve the mechanical properties. With the addition of 10 wt% compatibilizer, the elongation at break increased to 352.8%. As the concentration of compatibilizer increases, the crystallinity of PET decreases. The reaction of LLDPE-g-MAH with PET produces a PET-co-LLDPE-g-MA copolymer, which combines the interface chemically and improves the compatibility. Jayanarayanan et al. [13]. prepared LDPE/PET blends with LDPE as the continuous phase. By adding a compatibilizer, i.e., polyethylene grafted maleic anhydride (PE-g-MAH), under two matrix ratios, they found that it can reduce the particle size of PET and make it more evenly dispersed.

Regarding investigations of manufacturing immiscible polymer blends, current studies mainly focus on the development of new high-efficiency compatibilizers to improve the compatibility of the immiscible systems. To date, the processing technology of polymer materials is still based on screw equipment governed by shear rheology. Few investigations have focused on processing by changing the flow field in the equipment to enhance the interfacial interaction between the incompatible systems. Recently, an innovative eccentric rotor extruder (ERE) based on the elongational rheology field [14,15,16] was developed by our group for the processing of immiscible polymers. The elongational rheology field produces a velocity gradient along the flow direction by the periodic volume change in the processing unit in the device. The material can be periodically compressed and released under the action of the eccentric rotor. Compared to the conventional twin-screw extruder (TSE) based on the shear flow field, ERE can obviously enhance the dispersing and mixing effect of the immiscible systems and improve the phase morphology of the blends during the processing [17,18]. The revolutionary processing means have pointed out a new direction for immiscible polymer blending.

In this paper, a new type of ERE is employed to prepare the immiscible m-PE/PET blend. The phase morphology, rheological properties, crystallization behavior, and mechanical properties of the fabricated blends were compared with those of the material processed using traditional TSE to study the effect of the elongational rheology on the structure and properties of the immiscible blends. In order to prove the strengthening effect of ERE, a preliminary experiment using the compatibilizer, ethylene-co-methyl acrylate-co-glycidyl methacrylate (E-MA-GMA) which is the most effective compatibilizer, was carried out to prepare blends using ERE and TSE as references.

## 2. Experimental Section

### 2.1. Materials

The raw materials, i.e., m-PE (1018HA, specific gravity of 0.92 g/cm^3^) and PET (FY1002, specific gravity of 1.38 g/cm^3^), were obtained from Exxon Mobil Inc. (Irvine, TX, USA) and Pan-Asia PET Resin Inc. (Guangzhou, China), respectively. The compatibilizer for polymer blends, i.e., ethylene-*co*-methyl acrylate-*co*-glycidyl methacrylate (E-MA-GMA), were purchased from Arkema Investment Co., Ltd. (Shanghai, China)

### 2.2. Equipment

**Figure 1:**
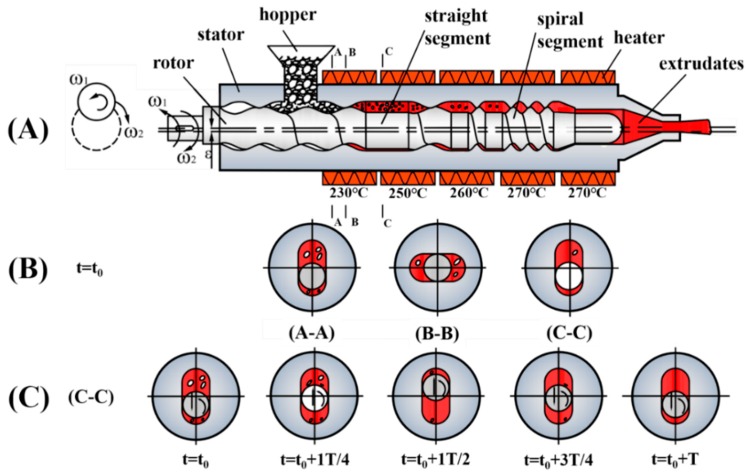
Schematic diagram of the self-developed eccentric rotor extruder (ERE). (**A**) the longitudinal section of ERE; (**B**) the rotor movement at different cross-section positions when *t* = *t*_0_; (**C**) the rotor movement in one cycle at cross-section C–C.

The ERE for the mixing of m-PE and PET to fabricate the m-PE/PET blends, as schematically shown in Figure 1a, was self-developed with an eccentricity (*ε*) of 5.0 mm. The eccentric rotor is divided into several alternating spiral segments and straight segments, and the interior of the stator is machined into the corresponding shape for a perfect engagement with the rotor (Figure 1b). During the processing of the material, the eccentric rotor rotates while revolving around the stator (Figure 1c). Figure 1c also shows the position and movement of the eccentric rotor when the eccentric rotor rotates within one rotation period. The polymer materials are delivered into the chamber between the rotor and stator when its volume increases, then plasticized and compacted under the elongational flow field. Thereafter, the polymer materials are melted, blended and finally extruded from the die. 

### 2.3. Sample Preparation

The raw materials, i.e., m-PE and PET, were dried at 120 °C for 8 h in a vacuum oven (DHG-9070, Yiheng, Shanghai, China) to completely remove the absorbed moisture before being processed. Subsequently, the dried m-PE and PET were melt blended with the aforementioned self-developed ERE device. The temperature of the extruder from hopper to die was kept at 230 °C–250 °C–260 °C–270 °C–270 °C as schematically shown in Figure 1a, and the rotating speed of the rotor was held at 30 rpm. The extrudate was pelletized to pellets and dried at 90 °C for 8 h in a vacuum oven to remove the absorbed moisture before being injection-molded into standard samples by an all-electric injection molding machine (105 GE, Donghua, Dongguan, China).

During the sample preparation, the composition of the m-PE/PET blends was varied to investigate its influence on the properties of the achieved blends. In this investigation, the m-PE/PET blends with the same compositions were also processed by a conventional twin-screw extruder (HT-20, Nanjing Rubber and Plastics, Nanjing, China), and compared to those processed using ERE. The L/D ratio of twin-screw extruder is 40, co rotating and intermeshing. The temperature of the extruder from hopper to die was kept at 230 °C–250 °C–270 °C–270 °C–270 °C–265 °C–265 °C–265 °C, and the rotating speed of twin-screw extruder is held at 180 rpm, at which the residence time of material in the barrel is the same as that of ERE. In order to prove the enhancement of interfacial interaction by the physical elongational flow field during the material processing, an effective compatibilizer, E-MA-GMA, was added at different concentrations to the blends to improve the interface compatibility. The compositions of all the fabricated samples were tabulated in Table 1 for easy reference.

### 2.4. Characterizations

#### 2.4.1. Scanning Electronic Microscope (SEM)

A scanning electronic microscope (SEM, FEI Quatan 250, FEI, Hillsborough, OR, USA) was applied to observe the fracture surfaces of the m-PE/PET blends to study the morphology of the blends and the size distribution of the dispersed PET particles. The samples were soaked in liquid nitrogen for 40 min and then cryo-fractured to obtain the fractured surface. In order to improve the conductivity of the polymer surface, gold sputter-coating was applied on the fracture surface for about 20 s for the SEM observation. The imaging was carried out under an operating voltage of 5 kV. The software, Image Pro, was used to measure the particle size in the blends. The number-average particle size (*D_n_*) was calculated using Equation (1) [19]:

(1) $$D_{n}=\frac{\sum n_{i}D_{i}}{\sum n_{i}}$$

where *D_i_* and *n_i_*, respectively, are the particle size and number of particle in the *i* interval.

#### 2.4.2. Differential Scanning Calorimetry (DSC)

The thermal behavior of the formulated blends was investigated using a Netzsch DSC204 (Bavaria, Germany) differential scanning calorimeter under a nitrogen atmosphere. The samples, with a weight of 4–6 mg, were firstly heated up from 30 °C to 280 °C with a heating rate of 10 °C/min and held at 280 °C for 5 min, then cooled down to 30 °C with a cooling rate of 10 °C/min, and finally, heated up again from 30 °C to 280 °C with a heating rate of 10 °C/min [20]. 

#### 2.4.3. Dynamic Rheology Measurements

To study the rheological behavior of the polymer blends, dynamic rheology measurements were conducted using a rotational rheometer (MCR302, Anton Paar, Graz, Austria). All the tests were carried out at a temperature of 260 °C with scanning frequencies from 0.0628 rad/s to 628 rad/s. During the measurements, the strain amplitude was kept at 5% [21]. 

#### 2.4.4. Mechanical Measurements

The tensile tests of the fabricated polymer blends were carried out following the ISO 527-2:1993 using a universal testing machine (Instron 5566, Instron, Boston, MA, USA). A loading rate of 50 mm/min was adopted for the crosshead. At least 5 pieces of specimen for each sample were tested to obtain an averaged value with a standard deviation.

## 3. Results and Discussion

### 3.1. Morphology Analysis

**Figure 2:**
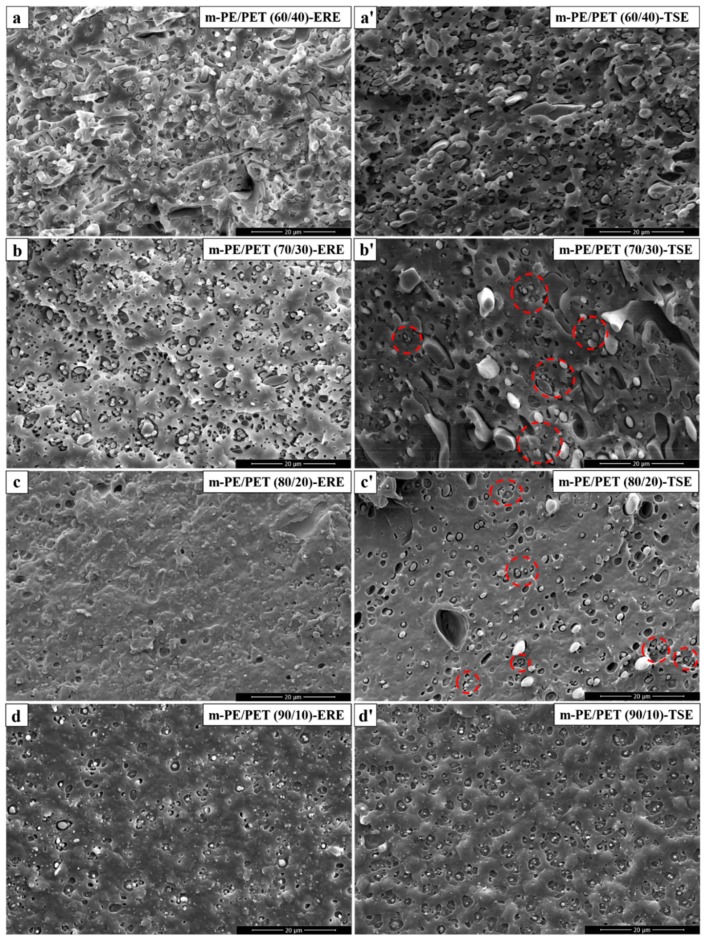
Scanning electron microscope (SEM) micrographs of the fabricated m-PE/PET blends processed using ERE and TSE, respectively. ERE processed blends: (**a**) m-PE/PET 60/40; (**b**) m-PE/PET 70/30; (**c**) m-PE/PET 80/20; (**d**) m-PE/PET 90/10. TSE processed blends: (**a’**) m-PE/PET 60/40; (**b’**) m-PE/PET 70/30; (**c’**) m-PE/PET 80/20; (**d’**) m-PE/PET 90/10.

The microstructure of the fabricated m-PE/PET blends was observed by SEM and is shown in Figure 2. As can be seen from the cryo-fractured surface, the PET phase is much more uniformly dispersed and the interface is much better in the blends processed using ERE, compared to those of the blends processed using TSE. From the analysis of the quenched section, the surface of the sample prepared using ERE is smooth and the interfacial gap between the two phases is smaller, while the surface of the sample prepared using TSE has many voids, indicating that the compatibility of the interface is relatively poor. In addition, evident agglomeration of the dispersed PET particles was observed in the blends prepared using TSE. The difference between the phase morphology of the samples prepared by the two devices is especially obvious at the matrix ratio of 80/20, as shown in Figure 2c,c’. Under the action of elongational rheology, the incompatible components become compatible to some degree, and no evident interface can be observed on the fracture surface.

**Figure 3:**
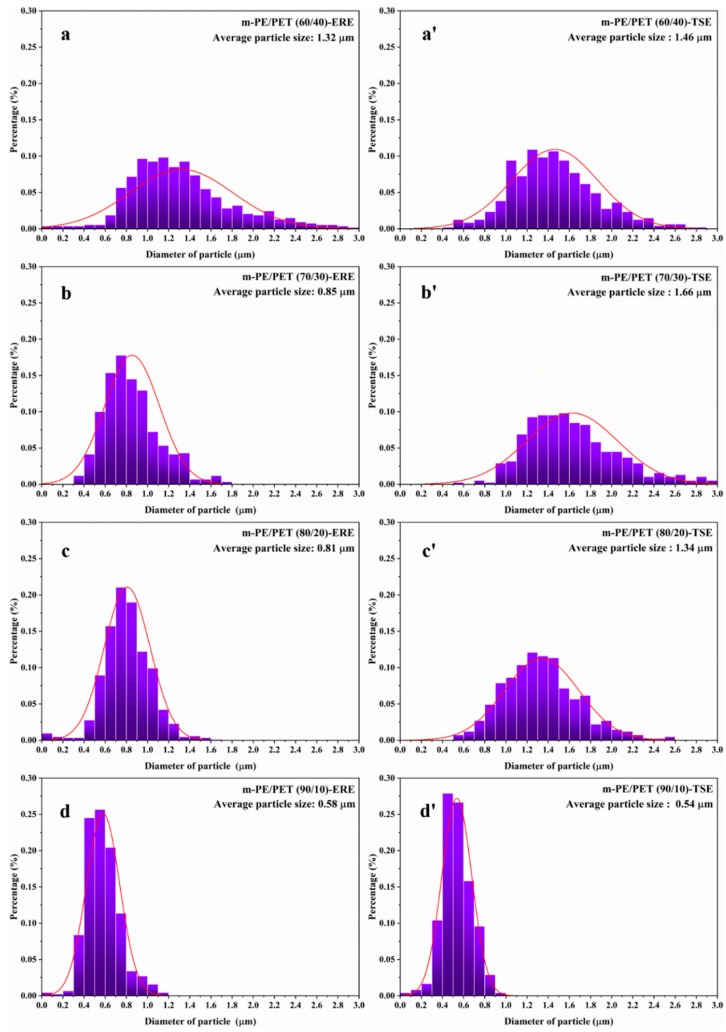
Statistics of the particle size and the size distribution of m-PE/PET blends processed using TSE and ERE, respectively. ERE processed blends: (**a**) m-PE/PET 60/40; (**b**) m-PE/PET 70/30; (**c**) m-PE/PET 80/20; (**d**) m-PE/PET 90/10. TSE processed blends: (**a’**) m-PE/PET 60/40; (**b’**) m-PE/PET 70/30; (**c’**) m-PE/PET 80/20; (**d’**) m-PE/PET 90/10.

As is known, the morphology, including the particle size and the size distribution of the dispersed phase in the matrix have a significant effect on the mechanical behavior of the material. In order to achieve the particle size and the size distribution of the blends, the particle size of PET in the m-PE/PET matrix with different compositions processed via ERE and TSE was measured using a software Image Pro, based on 300–400 counts that were randomly selected from at least 5 SEM images, as shown in Figure 3. It can be seen that the PET particles in the blends prepared using ERE is smaller and more uniform than that prepared using TSE. Except for the 90/10 matrix ratio, the PET particle size of the samples prepared using ERE at other matrix ratios is much smaller than that processed using TSE. Thus, this proves that the elongational rheology field is beneficial to the mixing and dispersing of immiscible polymer blends. 

**Figure 4:**
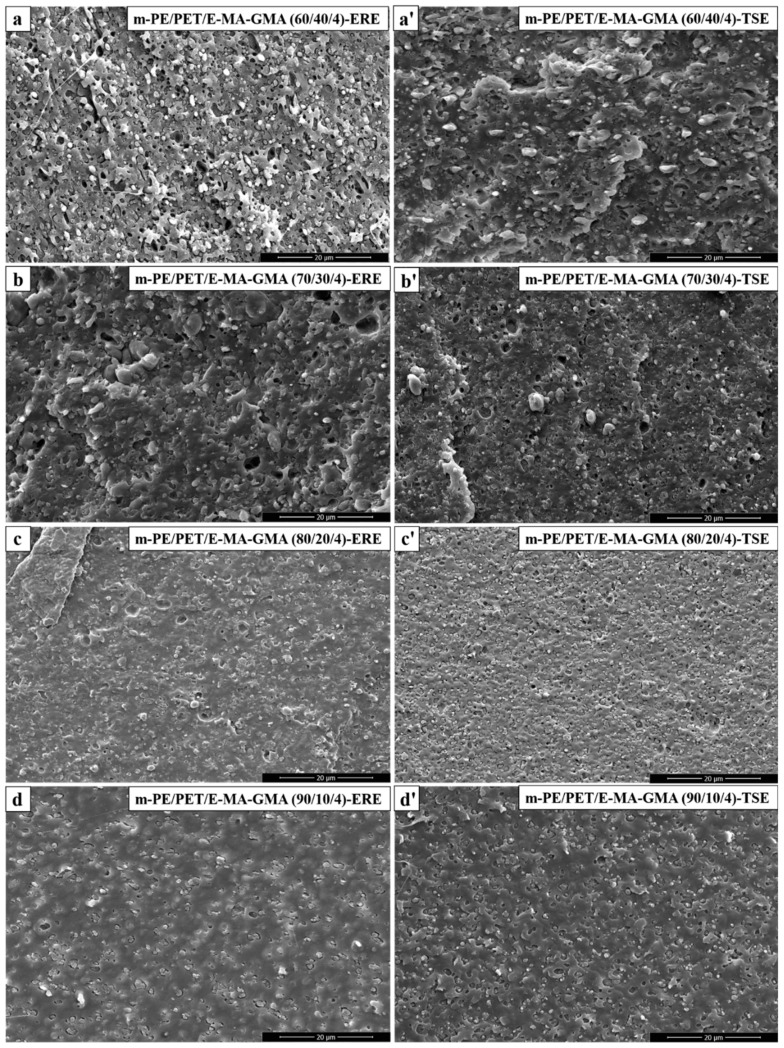
SEM micrographs of m-PE/PET/E-MA-GMA blends processed via ERE and TSE, respectively. ERE processed blends: (**a**) m-PE/PET/E-MA-GMA 60/40/4; (**b**) m-PE/PET/E-MA-GMA 70/30/4; (**c**) m-PE/PET/E-MA-GMA 80/20/4; (**d**) m-PE/PET/E-MA-GMA 90/10/4. TSE processed blends: (**a’**) m-PE/PET/E-MA-GMA 60/40/4; (**b’**) m-PE/PET/E-MA-GMA 70/30/4; (**c’**) m-PE/PET/E-MA-GMA 80/20/4; (**d’**) m-PE/PET/E-MA-GMA 90/10/4.

**Figure 5:**
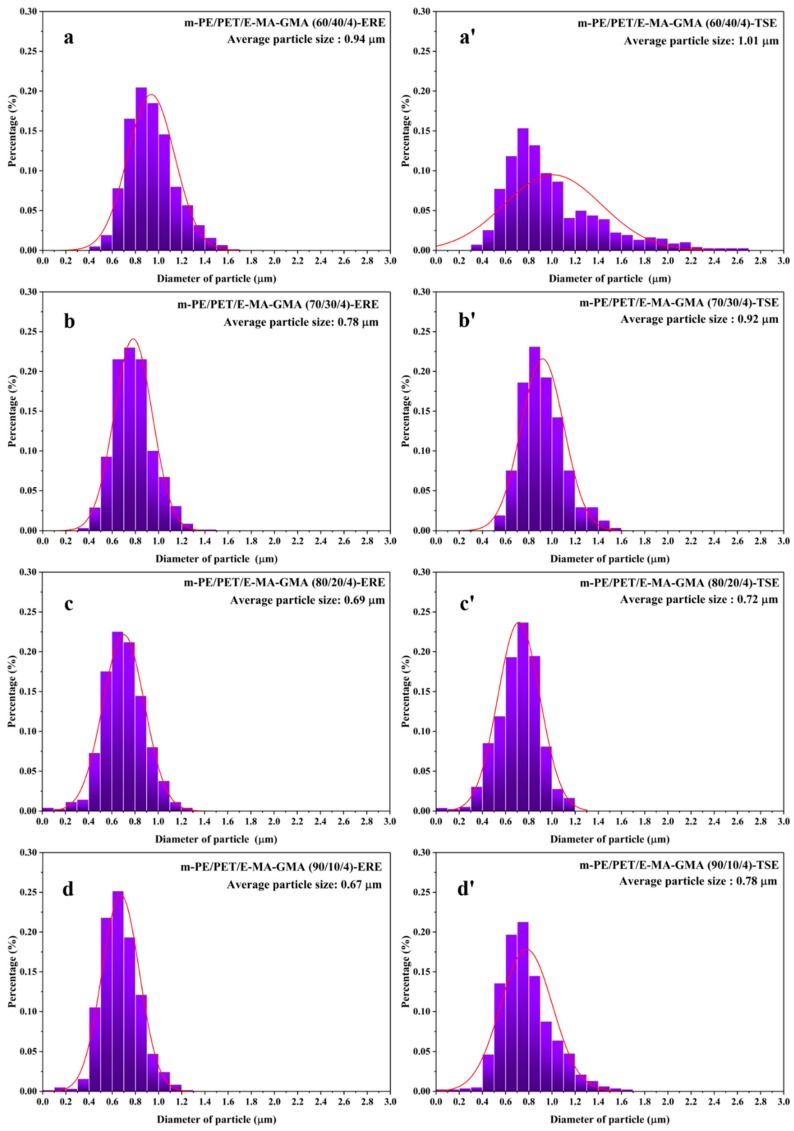
Statistics of particle size and size distribution of m-PE/PET/E-MA-GMA blends processed via ERE and TSE, respectively. ERE processed blends: (**a**) m-PE/PET/E-MA-GMA 60/40/4; (**b**) m-PE/PET/E-MA-GMA 70/30/4; (**c**) m-PE/PET/E-MA-GMA 80/20/4; (**d**) m-PE/PET/E-MA-GMA 90/10/4. TSE processed blends: (**a’**) m-PE/PET/E-MA-GMA 60/40/4; (**b’**) m-PE/PET/E-MA-GMA 70/30/4; (**c’**) m-PE/PET/E-MA-GMA 80/20/4; (**d’**) m-PE/PET/E-MA-GMA 90/10/4.

**Figure 6:**
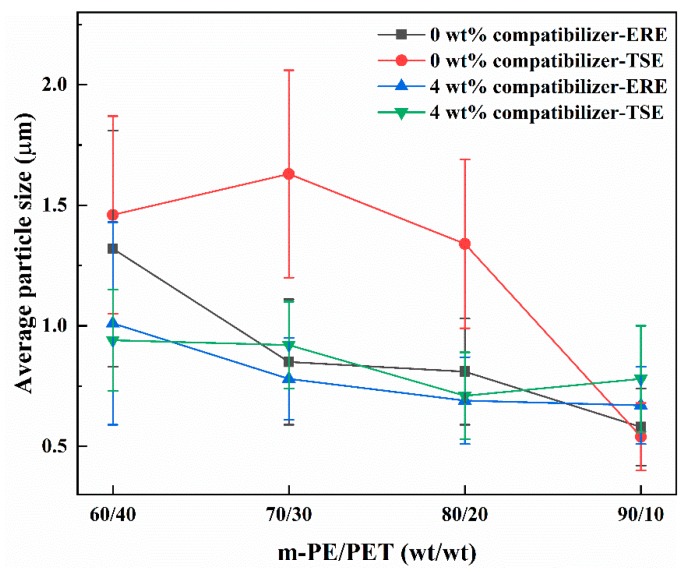
Average particle size of m-PE/PET blends (0 wt% and 4.0 wt% compatibilizer loaded) processed using ERE and TSE, respectively.

The m-PE/PET blends were added to 4.0 wt% E-MA-GMA as the phase compatibilizer and processed using ERE and TSE. Figure 4 shows the morphology of the cryo-fractured m-PE/PET blends with the compatibilizer, and Figure 5 shows the size distribution of the PET particles in the m-PE/PET blends. It can be seen that the compatibilizer can promote the dispersion of the PET phase in the m-PE matrix under the action of both the shear flow field and the elongation flow field, and that the blends processed by ERE have a better bonding at the interface. Although the size of the PET particle is smaller after the addition of the 4.0 wt% E-MA-GMA under both flow fields, the effect of the compatibilizer is more evident under the action of the shear flow field in the TSE. On the other hand, in comparison with the blends with 4.0 wt% compatibilizer processed using both equipment, perfect phase morphology, and particle size distribution can be achieved under the effect of an ERE without the compatibilizer, as shown in Figure 2, Figure 4 and Figure 6. The morphology and particle distribution are even better than that of the sample prepared by TSE with the addition of 4.0 wt% E-MA-GMA. The effect by adding E-MA-GMA on the ERE processed samples is not evident, which indicates that ERE itself has a certain capability to enhance compatibility. All of the above indicates that the elongational flow field in the ERE is beneficial to the dispersing and mixing of PET in the m-PE matrix without the addition of compatibilizers.

### 3.2. Differential Scanning Calorimetry (DSC) Test

**Figure 7:**
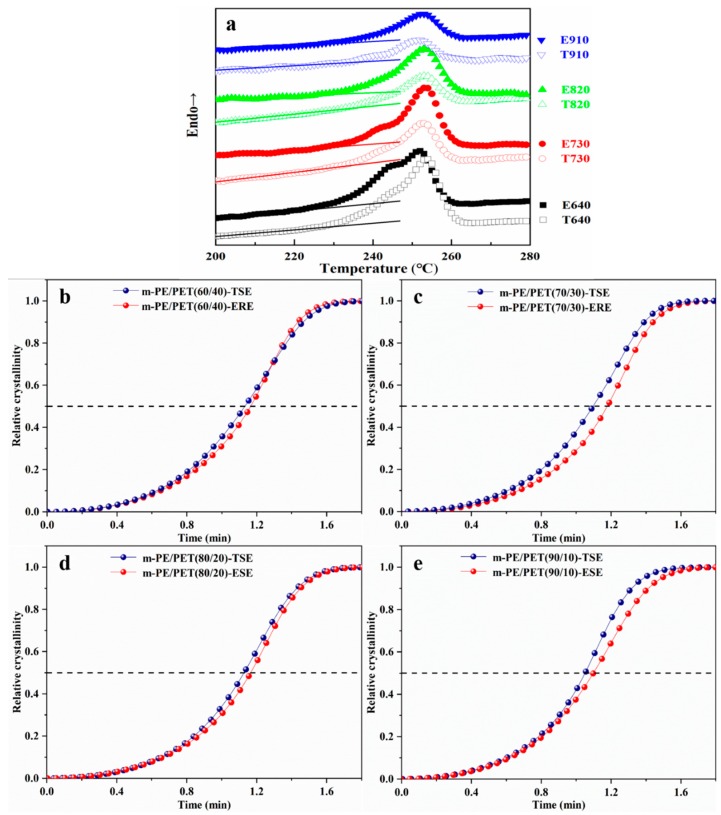
Second heating curve and the relative crystallinity of PET in m-PE/PET blends processed via ERE and TSE, respectively. (**a**) second-endothermic curves of the blends processed via ERE and TSE; (**b**–**e**) the relative crystallinity of the blends processed via ERE and TSE at different matrix ratio.

A DSC test was conducted to better understand the effect of the elongational flow field on the particle dispersion. The relative crystallinity of the PET in the m-PE/PET blends with respect to temperature can be derived from the DSC curve by integrating the DSC curve. Second-endothermic curves of blends were used to describe the crystallization behavior of PET, as shown in Figure 7a. It can be seen from the figure that PET in the ERE processed blends exhibited a lower initial melt temperature compared to that prepared by TSE, which signifies the lower initial crystallization temperature. The reason for this may be ascribed to the greater number of particles due to the excellent dispersion effect of ERE, thereby facilitating heterogeneous nucleation [19]. 

In order to achieve the Avrami equation, that is, the relative crystallinity (*X_t_*) as a function of time (*t*), the time-temperature conversion was performed using an equation as follows [22,23,24]:

(2) $$t=\frac{T_{0}-T}{\varphi }$$

where *T*_0_ is the temperature of initial crystallization, *T* is the crystallization temperature, and *t* is the crystallization time at which the crystallization temperature is *T*, *φ* is the drop rate of temperature. After conversion, the relationship between the relative crystallinity of the sample and the crystallization time under non-isothermal crystallization conditions can be obtained. Figure 7b–e show the relative crystallinity of PET in m-PE/PET blends processed using TSE and ERE. It can be seen that PET in the ERE processed blends showed a longer time for half crystallization (*t*_1/2_), i.e., the time required for achieving half of the final crystallinity. This indicates that the ERE process blends have a relatively slower crystallization rate at each matrix ratio. This phenomenon was caused by the excellent compatibilization between the two phases introduced by the elongational flow field during the material processing. Firstly, according to the foregoing result, the elongational flow field in the ERE is able to disperse the discontinuous PET phase more uniformly in the m-PE/PET blends with a much smaller particle size, compared to the shear flow field in the TSE. Since the particle size of the PET is at the micron or even sub-micron scale, the surface effect of the particle will be evident. Owing to the restriction of the surface, it is more difficult for the smaller particles to form ordered structures, resulting in a slow rate of crystallization and a long crystallization time. Secondly, the interdiffusion of immiscible polymers into each other at the interface can be enhanced by the elongational flow field, according to the literature [25]. Because of this, the chain entanglement of the two immiscible polymers is more serious at the interface, leading not only to the strengthening of the interface, but also to more difficulty in forming an ordered structure, i.e., crystallization, for the dispersed PET phase. 

### 3.3. Dynamic Rheology Properties

**Figure 8:**
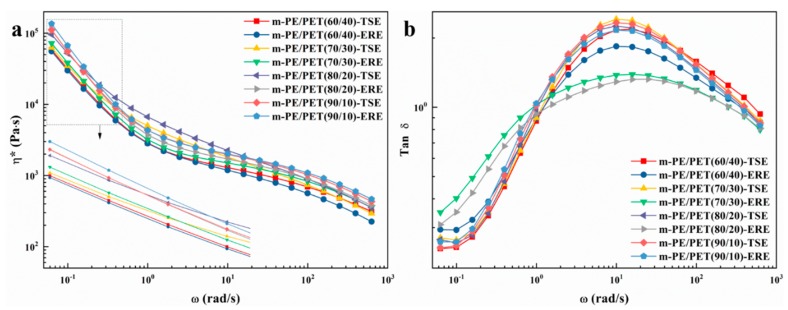
Dynamic rheology properties of m-PE/PET blends processed using ERE and TSE, respectively. (**a**) the complex viscosity of the blends processed by ERE and TSE; (**b**) the loss factor Tan δ of the blends processed by ERE and TSE.

The rheological behavior of the fabricated m-PE/PET blends was investigated using the dynamic rheological technique, which can indicate the interfacial compatibility [26,27,28,29,30] between two immiscible phases. Figure 8 shows the complex viscosity versus frequency for the m-PE/PET blends processed using TSE and ERE. As can be seen from Figure 8, the complex viscosity of the blends processed by ERE is higher than that processed by TSE. The complex viscosity of blends processed by ERE keeps at a higher level, indicating that the entanglement of the molecular chain is tighter under the action of the elongational flow field. The loss factor Tan δ (G″/G′) can reflect the viscoelastic response of the blends. It can be seen from Figure 8b that the blends processed via ERE has a smaller loss tangent peak at each matrix ratio. The smaller loss tangent peak indicates a lower loss modulus (G″) but a better elastic response (G′). While the former means the internal energy loss is lower due to less slip between the two immiscible phases under the external force, the latter indicates that the molecular entanglement is tight and the interface compatibility is better. Thus, it can be concluded that the elongational flow field is able to promote compatibility between the two immiscible phases by strengthening the interfacial interaction physically.

### 3.4. Mechanical Properties

**Figure 9:**
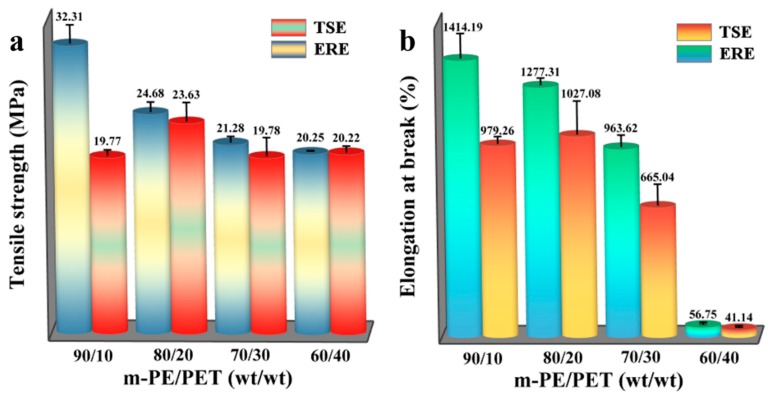
Tensile strength and elongation at break of m-PE/PET blends processed using ERE and TSE, respectively. (**a**) the tensile strength of the blends processed using ERE and TSE; (**b**) the elongation at break of the blends processed using ERE and TSE.

To further verify the effect of the elongational flow field in ERE on the promotion of mixing and the strengthening of the interface of the two immiscible polymers, the mechanical properties of the final blends were characterized. Figure 9 shows the tensile properties of the *m*-PE/PET blends processed using TSE and ERE. The tensile strength of the blends prepared using ERE is higher than that prepared using TSE for the samples with different compositions. When the matrix ratio is 90/10, the difference in tensile strength is particularly evident since the tensile strength of the blend increases by about 63% compared to that processed using TSE. For the elongation at break, it exhibited a similar trend to the tensile strength. ERE processed blends possess better properties at each matrix ratio. Particularly, the elongation at break of the blends (80/20 and 90/10) processed using ERE was improved by about 24% and 44% compared to that of the blends with the same compositions processed using TSE. The results are in accordance with the morphology change tendency. Due to the strengthened interface between the two immiscible polymers, and good dispersion of the PET phase in the m-PE matrix, ERE processed m-PE/PET blends exhibit better mechanical properties.

**Figure 10:**
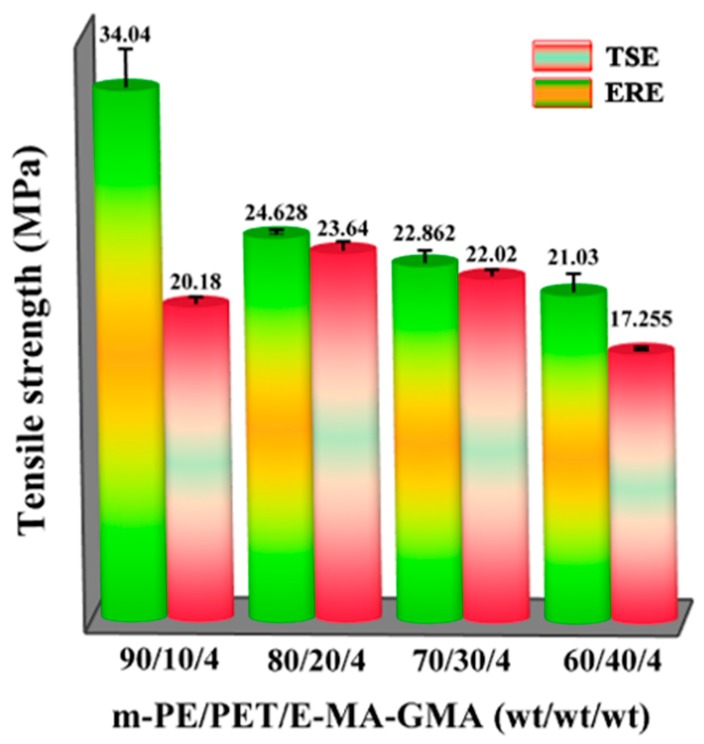
Tensile strength of m-PE/PET blends (4.0 wt% compatibilizer loaded) processed using ERE and TSE, respectively.

Figure 10 shows the trend of the tensile strength with respect to the matrix ratio of the m-PE/PET blends after the addition of 4.0 wt% compatibilizer. It can be seen that the blends with the compatibilizer prepared using both TSE and ERE shows a relatively higher tensile strength, compared to that without the compatibilizer as shown in Figure 9a. However, in all the matrix ratio of the m-PE/PET blends, the effect of the added compatibilizer is much more evident on the tensile strength of the blends processed by TSE, compared to that by ERE. This indirectly proves that the tensile strength of the ERE processed m-PE/PET blends is relatively high, even without the addition of any compatibilizers. Thus, the effect of the added compatibilizer on the tensile strength of the m-PE/PET blends is marginal when ERE was adopted.

For the 70/30 and 80/20 components, although there is little difference in mechanical properties between the samples processed by ERE and TSE, the average value of the ERE processed blends is higher than that processed by TSE, and the ERE processed blends have a smaller standard deviation, which shows that the two immiscible polymers are more evenly dispersed, that is to say ERE has a better compatibilization effect. As for 90/10 components, the results of complex viscosity and loss factor show that the ERE processed blends have a better compatibility, and the better compatibility brings better stress transfer to improve the performance. It is worth noting that the m-PE/PET blends without compatibilizer processed via ERE exhibit higher mechanical properties compared to that with 4.0 wt% compatibilizer processed using TSE, which further demonstrates the benefit of the elongational flow field in ERE during the processing of immiscible polymers.

## 4. Conclusions

In this investigation, a self-developed novel ERE was employed to process the immiscible m-PE and PET with different matrix ratios. The phase morphology, rheological properties, crystallization behavior, and mechanical properties of the fabricated blends were carefully studied to explore the effect of the elongational flow field in the ERE on the dispersion and mixing of PET in the m-PE matrix and the interface interaction of the two immiscible polymers. Compared to the same blend processed using TSE, the blend processed using ERE has a smaller size and a narrower size distribution of the PET particles in the m-PE matrix and better interface adhesion between the two immiscible polymers. This shows that the elongational flow field in ERE is much more effective at dispersing the immiscible PET in the *m*-PE matrix and strengthening the interface. For comparison, a compatibilizer, E-MA-GMA, was added to the m-PE/PET blends during the processing using TSE and ERE. Although the blends processed using both types of equipment show an improved mechanical property, the enhancement by adding the compatibilizer is marginal for the m-PE/PET blends processed using ERE, which indirectly proves the high effectiveness of the elongational flow field on the enhancement of the dispersion and mixing of PET in the m-PE matrix and the interface interaction. All these results demonstrated that ERE is effective equipment to process the immiscible polymer blends with high efficiency for the dispersion and mixing of the blends and strengthening the interface, and thus improves the mechanical properties.

## Figures and Tables

**Table 1:** Sample information of the fabricated metallocene polyethylene/poly(ethylene terephthalate) (m-PE/PET) blends in this investigation.

Sample	Composition		Compatibilizer ^a^ (wt%)	Processing Method
	m-PE (wt%)	PET (wt%)		
m-PE/PET(60/40)-ERE	60	40	-	ERE ^b^
m-PE/PET(70/30)-ERE	70	30	-	ERE
m-PE/PET(80/20)-ERE	80	20	-	ERE
m-PE/PET(90/10)-ERE	90	10	-	ERE
				
m-PE/PET(60/40)-TSE	60	40	-	TSE ^c^
m-PE/PET(70/30)-TSE	70	30	-	TSE
m-PE/PET(80/20)-TSE	80	20	-	TSE
m-PE/PET(90/10)-TSE	90	10	-	TSE
				
m-PE/PET/E-MA-GMA(60/40/4)-ERE	60	40	4.0	ERE
m-PE/PET/E-MA-GMA(70/30/4)-ERE	70	30	4.0	ERE
m-PE/PET/E-MA-GMA(80/20/4)-ERE	80	20	4.0	ERE
m-PE/PET/E-MA-GMA(90/10/4)-ERE	90	10	4.0	ERE
				
m-PE/PET/E-MA-GMA(60/40/4)-TSE	60	40	4.0	TSE
m-PE/PET/E-MA-GMA(70/30/4)-TSE	70	30	4.0	TSE
m-PE/PET/E-MA-GMA(80/20/4)-TSE	80	20	4.0	TSE
m-PE/PET/E-MA-GMA(90/10/4)-TSE	90	10	4.0	TSE

^a^ The adopted compatibilizer is ethylene-co-methyl acrylate-co-glycidyl methacrylate (E-MA-GMA); ^b^ ERE is the eccentric rotor extruder; ^c^ TSE is the twin-screw extruder.

## References

[1] Nitta K., Suzuki K., Tanaka A. (2000). Comparison of tensile properties in the pre-yield region of metallocene-catalyzed and Ziegler-Natta-catalyzed linear polyethylene. J. Mater. Sci..

[2] Kennedy M.A., Peacock A.J., Failla M.D., Lucas J.C., Mandelkern L. (1995). Tensile Properties of Crystalline Polymers: Random Copolymers of Ethylene. Macromolecules.

[3] Utracki L.A. (2002). Polymer Blends Hand Book.

[4] Dimitrova T.L., La Mantia F.P., Pilati F., Toselli M., Valenza A., Visco A. (2000). On the compatibilization of PET/HDPE blends through a new class of copolyesters. Polymer.

[5] Xanthos M., Dagli S. (1991). Compatibilization of polymer blends by reactive processing. Polym. Eng. Sci..

[6] Kalfoglou N.K., Skafidas D.S., Kallitsis J.K., Lambert J.C., Van der Stappen L. (1995). Comparison of Compatibilizer effectiveness for PET/HDPE blends. Polymer.

[7] Xue B., He H.Z., Huang Z.X., Zhu Z.W., Xue F., Liu S.M., Liu B. (2019). Fabrication of super-tough ternary blends by melt compounding of poly(lactic acid) with poly(butylene succinate) and ethylene-methyl acrylate-glycidyl methacrylate. Compos. Part B Eng..

[8] Xue B., He H.Z., Zhu Z.W., Li J.Q., Huang Z.X., Wang G.Z., Chen M., Zhan Z.M. (2018). A Facile Fabrication of High Toughness Poly (lactic Acid) via Reactive Extrusion with Poly (butylene Succinate) and Ethylene-Methyl Acrylate-Glycidyl Methacrylate. Polymers.

[9] Pracella M., Rolla L., Chionna D., Galeski A. (2002). Compatibilization and properties of poly (ethylene terephthalate)/polyethylene blends based on recycled materials. Macromol. Chem. Phys..

[10] Zhang H., Guo W., Yu Y., Li B., Wu C. (2007). Structure and properties of compatibilized recycled poly (ethylene terephthalate)/linear low density polyethylene blends. Eur. Polym. J..

[11] Zhang H., Zhang Y., Guo W., Xu D., Wu C. (2008). Thermal Properties and Morphology of Recycled Poly (ethylene terephthalate)/Maleic Anhydride Grafted Linear Low-Density Polyethylene Blends. J. Appl. Polym. Sci..

[12] Zhang Y., Guo W., Zhang H., Wu C. (2009). Influence of chain extension on the compatibilization and properties of recycled poly (ethylene terephthalate)/linear low density polyethylene blends. Polym. Degrad. Stab..

[13] Jayanarayanan K., Ravichandran A. (2010). Morphology and Mechanical Properties of Normal Blends and In-Situ Microfibrillar Composites from Low-Density Polyethylene and Poly (ethylene terephthalate). Polym-Plast Technol..

[14] Qu J.P. (2008). Polymer Plasticating and Conveying Method and Equipment based on Elongational Rheology. CN Patent.

[15] Qu J.P., Yang Z.T., Yin X.C., He H.Z., Feng Y.H. (2009). Characteristics Study of Polymer Melt Conveying Capacity in Vane Plasticization Extruder. Polym-Plast Technol..

[16] Qu J., Zhang N., Yu X., Zhang G., Liu S., Tan B., Liu L. (2013). Experimental Investigation of Polymer Pellets Melting Mechanisms in Vane Extruders. Adv. Polym. Technol..

[17] Qu J.P., Chen H.Z., Liu S.R., Tan B., Liu L.M., Yin X.C., Liu Q.J., Guo R.B. (2012). Morphology study of immiscible polymer blends in a vane extruder. J. Appl. Polym. Sci..

[18] Wu Z.H., Zhao Y.Q., Zhang G.Z., Yang Z.T., Qu J.P. (2013). Multifractal analysis on dispersion of immiscible high-density polyethylene/polystyrene blends processed via polymer vane plasticizing extruder. J. Appl. Polym. Sci..

[19] Chen Y., Fan J., Wang W., Wang Y., Xu C., Yuan D. (2017). Influence of size reduction of crosslinked rubber particles on phase interface in dynamically vulcanized poly (vinylidene fluoride)/silicone rubber blends. Polym. Test..

[20] Cock F., Cuadri A.A., García-Morales M., Partal P. (2013). Thermal, rheological and microstructural characterisation of commercial biodegradable polyesters. Polym. Test..

[21] Zou W., Chen R., Zhang G., Zhang H., Qu J. (2016). Mechanical, thermal and rheological properties and morphology of poly (lactic acid)/poly (propylene carbonate) blends prepared by vane extruder. Polym. Adv. Technol..

[22] Avrami M. (1939). Kinetics of phase change. I general theory. J. Chem. Phys..

[23] Ozawa T. (1971). Kinetics of non-isothermal crystallization. Polymer.

[24] Ziabicki H.A. (1978). Theoretical analysis of oriented and nonisothermal crystallization. Colloid Polym. Sci..

[25] He Y., Yang Z.T., Qu J.P. (2019). Super-toughed poly(lactic acid)/thermoplastic poly(ether)urethane nanofiber composites with in-situ formation of aligned nanofibers prepared by an innovative eccentric rotor extruder. Compos. Sci. Technol..

[26] Song Y., Zheng Q. (2016). Concepts and conflicts in nanoparticles reinforcement to polymers beyond hydrodynamics. Prog. Mater. Sci..

[27] Song Y., Zheng Q. (2016). A Guide for Hydrodynamic Reinforcement Effect in Nanoparticle-filled Polymers. Crit. Rev. Solid State Mater. Sci..

[28] Kiziltas A., Nazari B., Gardner D.J., Bousfield D.W. (2014). Polyamide 6–Cellulose Composites: Effect of Cellulose Composition on Melt Rheology and Crystallization Behavior. Polym. Eng. Sci..

[29] Chatterjee T., Krishnamoorti R. (2013). Rheology of polymer carbon nanotubes composites. Soft Matter..

[30] Parent J.S., Bodsworth A., Sengupta S.S., Kontopoulou M., Chaudhary B.I., Poche D., Cousteaux S. (2009). Structure-rheology relationships of long-chain branched polypropylene: Comparative analysis of acrylic and allylic coagent chemistry. Polymer.

